# Evaluating the efficacy of perioperative bundle care in reducing postoperative complications among patients undergoing coronary artery interventions

**DOI:** 10.1097/MD.0000000000043830

**Published:** 2025-10-10

**Authors:** Gaoya Li, Piaopiao Tan, Lu Li, Huarong Tan

**Affiliations:** a Department of Cardiovascular Medicine Center, The Central Hospital of Enshi Tujia and Miao Autonomous Prefecture, Enshi, Hubei Province, China; b Department of Radiology, The Central Hospital of Enshi Tujia and Miao Autonomous Prefecture, Enshi, Hubei Province, China.

**Keywords:** bundled care, patient satisfaction, percutaneous coronary intervention (PCI), postoperative complications, rehabilitation, retrospective study

## Abstract

This study sought to assess whether implementing a perioperative bundle care strategy could enhance recovery and minimize adverse events in patients undergoing percutaneous coronary intervention (PCI). A retrospective cohort analysis was conducted on 134 PCI patients treated between February 2023 and February 2024. Participants were categorized into 2 groups: an experimental group (n = 51) receiving structured bundle care and a control group (n = 83) managed with conventional protocols. Following propensity score matching, 50 patients from each cohort were included for comparative evaluation. Key metrics analyzed included baseline demographics, perioperative management, and postoperative outcomes, such as complication incidence, recovery progression, and patient-reported satisfaction. The bundle care intervention significantly lowered the occurrence of postoperative adverse events, including bleeding, hematoma, and pseudoaneurysm formation (*P* < .05). Additionally, this approach accelerated clinical recovery, evidenced by quicker stabilization of hemodynamic parameters (e.g., blood pressure and heart rate) and improved adherence to postoperative guidelines. Patients receiving bundle care also reported higher satisfaction levels and better health-related quality of life (HRQoL), alongside notable reductions in anxiety and depression scores compared to the control group. Perioperative bundle care demonstrates substantial benefits in PCI patients, including fewer complications, expedited recovery, and greater patient satisfaction. These findings suggest that this systematic, multidisciplinary approach holds significant potential for optimizing perioperative management in coronary interventions. Further prospective research is recommended to validate these results and investigate the long-term impact of bundle care in this clinical setting.

## 1. Introduction

Coronary heart disease (CHD) is one of the leading health threats worldwide, with both its incidence and mortality rates continuing to rise globally.^[1]^ According to the World Health Organization, cardiovascular diseases lead to nearly 18 million deaths annually, with approximately half attributed to CHD. As China’s population ages and unhealthy lifestyles, such as high-fat diets, smoking, and lack of exercise, become more widespread, the prevalence of CHD is increasing year by year. This has become a major issue affecting residents’ quality of life and public health resources.^[2]^

In recent years, percutaneous coronary intervention (PCI), a minimally invasive treatment, has been widely adopted in the management of CHD.^[3]^ PCI rapidly restores coronary blood flow through mechanical dilation and stent implantation, significantly improving patients’ symptoms and cardiac function, making it a primary treatment for stable angina and acute coronary syndrome.^[4]^ However, there are notable limitations to PCI treatment: although vascular stenosis is corrected, restenosis and thrombosis within the stent remain significant complications. Additionally, postoperative patients often face long-term management issues regarding risk factors such as hypertension, hyperlipidemia, and diabetes.^[5]^ Therefore, optimizing perioperative management is crucial to enhancing the effectiveness of PCI and reducing postoperative adverse events.

Perioperative nursing plays an indispensable role in the comprehensive treatment of CHD patients. Through preoperative psychological interventions, intraoperative monitoring, and early postoperative rehabilitation guidance, perioperative care can not only help patients improve their understanding of the disease and surgery but also enhance their treatment adherence and quality of life.^[6]^ Additionally, preoperative care, as a key component of perioperative management, has a profound impact on the success of PCI and postoperative prognosis. Preoperative care helps comprehensively assess patients’ conditions, comorbidities, and risk factors, providing detailed information to support the surgical process. It also optimizes blood pressure, blood glucose, and cardiac function, thereby reducing the risk of intraoperative and postoperative complications.^[7]^ Furthermore, preoperative care includes psychological support and education, which can significantly alleviate patients’ anxiety, boost their confidence in the surgery, and promote adherence to medical advice, laying a solid foundation for long-term postoperative management.^[8]^

However, traditional nursing models often focus on single interventions, lacking systematic and comprehensive approaches, which cannot fully address the complex care needs of PCI patients. In contrast, bundle care is an integrated nursing model that incorporates multiple evidence-based measures. By systematically assessing patients’ individualized needs and optimizing the nursing process, bundle care combines preoperative education, intraoperative monitoring, postoperative rehabilitation, and follow-up management, aiming to achieve optimal care outcomes at the lowest cost.^[9]^ Bundle care has demonstrated significant clinical advantages in intensive care, postoperative rehabilitation, and chronic disease management, but its application and evaluation in the perioperative period of PCI require further study.

Based on the above context, this study aims to evaluate the application value of bundle care in the perioperative period of PCI for CHD patients. Specifically, we will use a retrospective study design to compare the differences in postoperative complication rates, rehabilitation quality, and nursing satisfaction between patients receiving bundle care and those receiving traditional care, exploring the role of bundle care in improving PCI patient outcomes, and providing scientific evidence for the further promotion and optimization of this nursing model.

## 2. Materials and methods

### 2.1. Study design

This study was approved by the Ethics Committee of the Central Hospital of Enshi Tujia and Miao Autonomous Prefecture. This retrospective study collected data from 134 patients who underwent PCI at our hospital between February 2023 and February 2024. Patients were divided into 2 groups based on their previous perioperative care approaches: the experimental group (51 patients who received bundle care) and the control group (83 patients who received routine care). To eliminate confounding factors, 50 matched patients were selected from each group based on baseline characteristics.

Inclusion criteria: diagnosed with coronary atherosclerotic heart disease (CHD) and undergoing PCI. Aged 18 years or older. Complete clinical data including preoperative assessments, nursing records, and postoperative complications. Follow-up time ≥ 30 days post-surgery.

Exclusion criteria: severe liver, kidney dysfunction, or other systemic diseases. Underwent other cardiovascular interventions or surgery within the last 6 months. Death or loss to follow-up for reasons unrelated to PCI. Patients with poor compliance to nursing interventions.

## 3. Nursing methods assessment

### 3.1. *Routine care*^[10]^

#### 3.1.1. Preoperative care

Nurses performed a comprehensive assessment of the patient, including history collection, monitoring vital signs, and verifying necessary preoperative tests (e.g., blood tests, coagulation profile, ECG). Detailed explanations of the surgery, its risks, and postoperative care were provided to the patient and family, obtaining informed consent while alleviating patient anxiety. The puncture site (e.g., radial or femoral artery) was cleaned and prepared to prevent infection. Patients with allergy histories received prescribed prophylactic antiallergy medications (e.g., diphenhydramine). The patient was instructed to fast for 8 hours before surgery and to avoid drinking fluids for 4 hours to ensure safety during the procedure.

#### 3.1.2. Intraoperative care

Nurses assisted the physician with local anesthesia, monitoring the patient’s tolerance to anesthetics (e.g., lidocaine), and assisted with identifying the puncture site. Key surgical details such as the number of affected vessels, lesion location, balloon inflation pressure, and stent type and placement were documented. Vital signs (heart rate, blood pressure, oxygen saturation) were continuously monitored, with prompt reporting of any abnormality. Hemostasis measures were implemented post-procedure, such as using radial artery hemostasis devices or manual compression, and the puncture site was checked for bleeding, hematoma, or pseudoaneurysm.

#### 3.1.3. Postoperative care

Post-surgery, nurses monitored the patient’s vital signs and puncture site for signs of complications like bleeding, hematoma, or pseudoaneurysm. The patient was encouraged to drink plenty of fluids to help excrete the contrast agent and was instructed to follow prescribed medications (e.g., aspirin, clopidogrel) for antiplatelet therapy. Strict bed rest was required (minimum of 6 hours for radial puncture and 12 to 24 hours for femoral puncture) to prevent complications, and the patient was educated about potential postoperative discomforts and the importance of follow-up visits and long-term medication adherence.

#### 3.1.4. Follow-up care

Nurses conducted regular follow-up through phone calls or outpatient visits to monitor the patient’s recovery progress, medication adherence (especially antiplatelet drugs), and any complications such as chest pain, shortness of breath, bleeding, or hematoma. Personalized health guidance was provided, including recommendations for diet, smoking cessation, alcohol reduction, and appropriate exercise, to reduce the risk of cardiovascular events and improve overall quality of life.

### 3.2. *Bundle care*^[11]^

Bundle care involves a more in-depth approach to perioperative care, incorporating comprehensive interventions before, during, and after the surgery.

#### 3.2.1. Preoperative psychological support

Psychological support in Bundle care is more detailed. Nurses begin by assessing patients’ anxiety levels using standardized tools like the self-rating anxiety scale (SAS). Based on their scores, patients are categorized into mild, moderate, or severe anxiety groups and receive targeted interventions. For mild anxiety, nurses use simple language to explain the surgery’s purpose, process, and successful outcomes, alleviating concerns. Moderate and severe anxiety patients receive one-on-one psychological counseling, focusing on their specific concerns, and are taught relaxation techniques, such as deep breathing or muscle relaxation. Family members are encouraged to participate, helping create a collaborative support network.

#### 3.2.2. Environmental intervention

Optimizing the hospital environment is a key component of Bundle care. Nurses adjust the room’s temperature (20°C–25°C) and humidity (40%–60%), ensuring air quality by introducing air purifiers. Soft lighting and calming background music (such as light instrumental music) are used to create a relaxing atmosphere. In terms of patient routines, a personalized schedule is developed based on the patient’s preferences. Nursing procedures like infusions and tests are scheduled during waking hours to minimize nighttime disturbances. If nighttime interventions are necessary, patients are informed in advance to reduce emotional distress.

#### 3.2.3. Intraoperative care

Intraoperative care in Bundle care enhances dynamic monitoring and psychological comfort. Nurses select appropriate hemostasis devices (e.g., radial artery compression devices or balloon hemostats) based on the puncture site and vascular conditions. Every 5 minutes, nurses record vital signs (heart rate, blood pressure, oxygen saturation), and any abnormalities are immediately communicated to the surgical team. Nurses also provide emotional support during the procedure by explaining the progress to the patient, soothing any anxiety, and offering comfort (e.g., gentle shoulder pats) if the patient experiences symptoms like chest pain or nausea.

#### 3.2.4. Postoperative puncture site management

Postoperative care focuses intensively on managing puncture sites. Nurses assess the radial artery puncture site for bleeding or hematoma every 2 hours and check the distal pulse. For femoral artery punctures, assessments are made every hour, observing for changes in skin color and temperature, and monitoring for potential complications like arteriovenous fistulas or pseudoaneurysms. High-risk patients undergo dynamic ultrasound to evaluate blood flow. Nurses also guide patients to avoid exertion on the puncture side and adhere strictly to prescribed bed rest times to prevent complications.

#### 3.2.5. Medication and hydration management

In Bundle care, medication and hydration management is highly personalized. Nurses adjust antiplatelet drugs (e.g., aspirin, clopidogrel) based on the patient’s liver and kidney function, weight, and coagulation profile, optimizing drug efficacy while minimizing risks. Hydration management is crucial, with nurses advising patients to drink 1500 to 2000 mL of fluids daily post-surgery and monitoring urine output. For patients with inadequate urine output or renal issues, intravenous fluids are administered at a rate of 100 to 200 mL/h, with regular monitoring of kidney function and electrolyte levels to facilitate the elimination of contrast agents.

#### 3.2.6. Health education

Health education in Bundle care is tailored and continually optimized. Nurses use multimedia tools like slides and animations to explain key postoperative care points, including puncture site care, proper use of antiplatelet medications, and early recognition of complications. Education is customized to address specific knowledge gaps in both patients and their families, with follow-up questions to assess understanding. Family members are involved in the entire educational process, ensuring they can monitor and care for the patient post-discharge, especially in the event of unexpected complications like chest pain or abnormal puncture site changes.

#### 3.2.7. Follow-up management

Follow-up care in Bundle care is highly personalized. Nurses develop a follow-up plan based on the patient’s risk level, scheduling more frequent follow-ups (e.g., by phone or outpatient visits) for high-risk patients. Standard patients are followed up monthly. During follow-up, nurses assess the patient’s recovery and monitor for postoperative complications. They also evaluate the patient’s exercise tolerance and provide progressive rehabilitation guidance. Additionally, tailored dietary recommendations and lifestyle adjustments are provided to ensure long-term health management.

## 4. 3. Data collection and indicator evaluation

### 4.1. Baseline characteristics

Key baseline characteristics include: age, gender, severity of coronary artery disease (CAD), health-related quality of life (HRQoL) total score^[12]^ and common comorbidities.

### 4.2. Preoperative psychological assessment

Anxiety level is assessed using the self-rating anxiety scale (SAS).^[13]^ Depression symptoms are evaluated using the Patient Health Questionnaire (PHQ)-9.^[14,15]^

### 4.3. Intraoperative indicators

Key intraoperative measures include: mean arterial pressure (MAP) and heart rate stability; adverse events, including: allergic reactions, bleeding at the puncture site, hematoma formation rate and surgical duration.

### 4.4. Postoperative indicators

Postoperative outcomes focus on: complications, such as: bleeding, hematomas, pseudoaneurysms, cardiovascular events and contrast-induced nephropathy (CIN). Recovery indicators, including: blood pressure and heart rate recovery times, urine output; patient compliance and quality of life.

## 5. Statistical analysis

For statistical analysis, propensity score matching (PSM) is used to reduce bias and balance baseline characteristics between groups. A 1:1 nearest-neighbor matching method is employed, using variables like age, gender, severity of CAD, and comorbid conditions to calculate the propensity score. A matching radius of 0.02 is set to maximize balance across groups.

Post-matching, differences in continuous variables are analyzed using: independent *t*-tests or Mann–Whitney *U* tests Categorical variables are compared using: Chi-square tests or Fisher exact tests odds ratios (OR) and 95% confidence intervals are also computed. For paired data, further analysis is done using: paired *t*-tests or Wilcoxon signed-rank tests In multivariable analyses, linear regression is used for continuous outcomes, and logistic regression for binary outcomes. Potential confounding factors are adjusted, and model fit is assessed using the Hosmer–Lemeshow test.^[16]^ Data analysis is conducted with SPSS 27.0 (Chicago) and R 4.2.3, with PSM performed using the MatchIt R package. A significance level of *P* < .05 is used, and matching balance is evaluated using the standardized mean difference (SMD < 0.1) to ensure scientific rigor.

## 6. Results

### 6.1. Propensity score matching: balance of key factors

After PSM, the experimental and control groups were balanced in terms of CAD severity scores (*P* = .521), indicating that matching eliminated baseline differences between groups. Before matching, the experimental group had a significantly higher CAD severity score (18.5 ± 4.3) compared to the control group (16.9 ± 4.1), suggesting a more severe baseline condition in the experimental group. After matching, both groups showed similar scores (experimental group: 18.3 ± 4.2; control group: 18.0 ± 4.4), Table 1.

**Table 1:** Comparison of baseline characteristics between the experimental group and the control group before and after propensity score matching.

Variables	Pre-match experimental group (n = 51)	Pre-match control group (n = 83)	Pre-match *P*-value	Post-match experimental group (n = 50)	Post-match control group (n = 50)	Post-match *P*-value
Age (year)	63.4 ± 8.2	65.7 ± 7.8	.045	63.5 ± 8.1	64.1 ± 8.2	.634
Gender (male)	37 (72.5%)	62 (74.7%)	.845	36 (72.0%)	35 (70.0%)	.812
Severity score of CAD	18.5 ± 4.3	16.9 ± 4.1	.022	18.3 ± 4.2	18.0 ± 4.4	.521
Hypertension	35 (68.6%)	55 (66.3%)	.715	34 (68.0%)	33 (66.0%)	.731
Diabetes mellitus	18 (35.3%)	31 (37.3%)	.782	17 (34.0%)	18 (36.0%)	.852
Smoking history	25 (49.0%)	42 (50.6%)	.846	24 (48.0%)	23 (46.0%)	.793
BMI (kg/m^2^)	25.4 ± 3.2	25.1 ± 3.0	.611	25.4 ± 3.1	25.3 ± 3.2	.843
Preoperative antiplatelet medication use	50 (98.0%)	81 (97.6%)	.894	49 (98.0%)	48 (96.0%)	.734
Radial access site	45 (88.2%)	72 (86.7%)	.734	44 (88.0%)	43 (86.0%)	.712
Number of stents implanted	2.2 ± 1.1	2.3 ± 1.0	.611	2.2 ± 1.0	2.3 ± 1.1	.653
High-risk factors for complications	21 (41.2%)	35 (42.2%)	.951	21 (41.0%)	22 (44.0%)	.794
HRQoL total score	85.1 ± 6.5	84.8 ± 6.8	.768	85.3 ± 6.4	85.2 ± 6.7	.891

BMI = body mass index, CAD = coronary artery disease, HRQoL = health-related quality of life.

Another key factor, health-related quality of life (HRQoL) total score, assessing physical, psychological, and social functional status, showed no significant difference between the groups prior to matching (experimental group: 85.1 ± 6.5; control group: 84.8 ± 6.8, *P* = .768). After matching, the HRQoL scores were also balanced (experimental group: 85.3 ± 6.4; control group: 85.2 ± 6.7, *P* = .891), further ensuring the accuracy of the intervention effect evaluation.

These results highlight the effectiveness of PSM in balancing key baseline characteristics between groups, enhancing the reliability of the study’s conclusions.

### 6.2. Comparison of preoperative psychological status

Preoperative psychological state significantly impacts surgery cooperation and recovery. This study assessed the preoperative anxiety and depression levels in both the experimental and control groups (Table 2). At admission, there were no significant differences in SAS (SAS: experimental group 58.2 ± 7.5, control group 57.8 ± 7.3, *P* = .803) and PHQ-9 scores (experimental group: 13.5 ± 3.2, control group: 13.2 ± 3.1, *P* = .638), indicating similar baseline conditions. After preoperative intervention, the experimental group showed significant improvement. The SAS score for the experimental group (45.3 ± 6.8) was significantly lower than the control group (50.1 ± 6.9, *P* < .001), and the PHQ-9 score was similarly improved (experimental group: 7.8 ± 2.9, control group: 10.4 ± 3.0, *P* < .001). These findings demonstrate that the psychological support component of Bundle care significantly reduced preoperative anxiety and depression in the experimental group.

**Table 2:** Comparison of anxiety and depression scores between the experimental group and the control group on admission and before operation.

Group	SAS score (admission)	SAS score (pre-surgery)	PHQ-9 score (admission)	PHQ-9 score (pre-surgery)
Experimental group (n = 50)	58.2 ± 7.5	45.3 ± 6.8	13.5 ± 3.2	7.8 ± 2.9
Control group (n = 50)	57.8 ± 7.3	50.1 ± 6.9	13.2 ± 3.1	10.4 ± 3.0
Statistical value	0.25	−3.65	0.47	−4.15
*P*-value	.803	<.001	.638	<.001

PHQ = Patient Health Questionnaire, SAS = self-rating anxiety scale.

## 7. Comparison of intraoperative vital signs and adverse events

Intraoperative vital signs and adverse events are critical in assessing the impact of Bundle care on safety, complication control, and surgical efficiency. As shown in Table 3, the experimental group had significantly lower MAP and heart rate compared to the control group (MAP: 85.3 ± 6.5 mm Hg vs 89.7 ± 7.2 mm Hg, *P* = .002; heart rate: 72.4 ± 8.3 bpm vs 76.2 ± 9.0 bpm, *P* = .037), indicating that Bundle care may help maintain more stable vital signs during surgery.

**Table 3:** Comparison of intraoperative vital signs, adverse events and operative time.

Group	Mean arterial pressure (mm Hg)	Heart rate (bpm)	Intraoperative allergic reactions (n, %)	Puncture site bleeding (n, %)	Puncture site hematoma (n, %)	Operation time (min)
Experimental group (n = 50)	85.3 ± 6.5	72.4 ± 8.3	1 (2.0%)	2 (4.0%)	1 (2.0%)	52.3 ± 10.5
Control group (n = 50)	89.7 ± 7.2	76.2 ± 9.0	4 (8.0%)	7 (14.0%)	6 (12.0%)	59.7 ± 12.3
Statistical value	*t* = −3.10	*t* = -2.12	χ² = 2.16	χ² = 3.09	χ² = 4.34	*t* = −3.02
*P*-value	.002	.037	.141	.079	.037	.004

Regarding intraoperative adverse events, the experimental group had lower rates of allergic reactions, puncture site bleeding, and hematoma formation compared to the control group. Although differences in allergic reactions and puncture site bleeding did not reach statistical significance (*P* = .141 and *P* = .079), the hematoma formation rate was significantly lower in the experimental group (*P* = .037). Additionally, the experimental group had a significantly shorter surgery time (52.3 ± 10.5 minutes vs 59.7 ± 12.3 minutes, *P* = .004). These results suggest that Bundle care may improve intraoperative stability, reduce puncture site complications, and optimize surgical efficiency.

## 8. Postoperative complications, recovery, and compliance

The experimental group showed lower rates of postoperative bleeding (4.0% vs 12.0%, *P* = .102), hematoma (2.0% vs 8.0%, *P* = .087), and pseudoaneurysm (0.0% vs 4.0%, *P* = .149), though the differences were not statistically significant. Cardiovascular event rates were also lower (2.0% vs 8.0%, *P* = .109), Table 4. Notably, CIN occurred less often in the experimental group (4.0%) compared to the control group (14.0%, *P* = .04).

**Table 4:** Comparison of postoperative complication rate and recovery speed.

Indicator	Experimental group (n = 50)	Control group (n = 50)	Statistical value	*P*-value
Bleeding incidence (n, %)	2 (4.0%)	6 (12.0%)	χ² = 2.67	.102
Hematoma incidence (n, %)	1 (2.0%)	4 (8.0%)	χ² = 2.93	.087
Pseudoaneurysm incidence (n, %)	0 (0.0%)	2 (4.0%)	χ² = 2.08	.149
Cardiovascular events (n, %)	1 (2.0%)	4 (8.0%)	χ² = 2.56	.109
CIN incidence (n, %)	2 (4.0%)	7 (14.0%)	χ² = 4.23	.04
BP recovery time (h)	8.5 ± 1.7	11.2 ± 2.1	t = -7.12	<.001
Heart rate recovery time (h)	7.8 ± 1.5	10.3 ± 1.9	t = -7.88	<.001
Urine output (mL/d)	2100 ± 350	1800 ± 300	t = 4.32	<.001
Antiplatelet tolerance adjustments (n, %)	3 (6.0%)	9 (18.0%)	χ² = 4.55	.033
Bed rest duration (h)	6.3 ± 1.2	7.5 ± 1.4	t = −4.24	<.001
Post-op mobility time (h)	24.8 ± 3.5	30.2 ± 4.0	t = -6.49	<.001

BP = blood pressure, CIN = contrast-induced nephropathy.

Recovery indicators revealed significant improvements for the experimental group: shorter blood pressure recovery time (8.5 ± 1.7 vs 11.2 ± 2.1 hours, *P* < .001) and heart rate recovery time (7.8 ± 1.5 vs 10.3 ± 1.9 hours, *P* < .001), along with higher urine output (2100 ± 350 vs 1800 ± 300 mL, *P* < .001). Additionally, the experimental group had fewer antiplatelet adjustments (6.0% vs 18.0%, *P* = .033), suggesting better medication adherence. The group also experienced shorter bed rest (6.3 ± 1.2 vs 7.5 ± 1.4 hours, *P* < .001) and activity recovery times (24.8 ± 3.5 vs 30.2 ± 4.0 hours, *P* < .001), indicating faster postoperative recovery.

## 9. Postoperative adverse events comparison

The comparison of postoperative adverse events between the experimental and control groups provides insight into the impact of different nursing interventions on reducing complications. As shown in Table 5, in patients requiring intervention for bleeding, the experimental group had 1 case (2.0%) while the control group had 3 cases (6.0%), though the difference was not statistically significant (*P* = .285). Regarding cardiovascular events leading to rehospitalization, 1 case (2.0%) occurred in the experimental group, compared to 2 cases (4.0%) in the control group, with no significant difference (*P* = .561). Infection rates were 0% in the experimental group versus 4.0% in the control group, but this difference was not statistically significant (*P* = .149). For patients with pseudoaneurysm requiring treatment, both groups had 1 case (2.0%), showing no significant difference (*P* = 1). Overall, while some adverse events were less frequent in the experimental group, the majority of differences did not reach statistical significance, indicating no substantial difference in the occurrence of these complications between the 2 groups.

**Table 5:** Comparison of postoperative adverse event types.

Adverse event type	Experimental group (n = 50)	Control group (n = 50)	Statistical value	*P*-value
Bleeding requiring intervention (n, %)	1 (2.0%)	3 (6.0%)	χ² = 1.14	.285
Rehospitalization for cardiovascular events (n, %)	1 (2.0%)	2 (4.0%)	χ² = 0.34	.561
Infection (n, %)	0 (0.0%)	2 (4.0%)	χ² = 2.08	.149
Pseudoaneurysm requiring treatment (n, %)	1 (2.0%)	1 (2.0%)	χ² = 0.00	1

## 10. Comparison of health-related quality of life dimensions between experimental and control groups

Health-related quality of life (HRQoL) is a key indicator for assessing postoperative recovery and the impact of nursing interventions. The HRQoL dimensions, including physical, psychological, and social functions, were compared between the experimental and control groups to assess the effects of bundled care. As shown in Table 6, the experimental group had significantly better scores in physical functioning, role functioning (RP), bodily pain, general health, vitality (VT), and overall quality of life compared to the control group (*P* < .001). However, no significant differences were observed between the 2 groups in social functioning, emotional role functioning (RE), and mental health dimensions (*P* > .05). These findings suggest that bundled care had a more pronounced effect on improving physical health and overall quality of life, while its impact on psychological and social aspects was less significant.

**Table 6:** Comparison of various dimensions of HRQoL scores between the experimental and control groups.

Dimension	Experimental group (n = 50)	Control group (n = 50)	Statistical value	*P*-value
PF	88.5 ± 6.4	81.2 ± 7.3	*t* = 5.29	<.001
RP	85.3 ± 8.2	78.5 ± 8.9	*t* = 4.37	<.001
BP	86.7 ± 7.8	79.8 ± 7.5	*t* = 4.55	<.001
GH	83.5 ± 6.9	77.1 ± 7.2	*t* = 4.71	<.001
VT	84.2 ± 7.1	75.3 ± 7.8	*t* = 5.81	<.001
SF	87.6 ± 6.5	87.4 ± 6.7	*t* = 0.12	.905
RE	85.1 ± 7.3	84.8 ± 7.5	*t* = 0.22	.826
MH	86.3 ± 6.8	86.1 ± 6.9	*t* = 0.18	.856
Overall HRQoL Score	85.6 ± 6.8	78.2 ± 7.5	*t* = 5.27	<.001

BP = bodily pain, GH = general health, HRQoL = health-related quality of life, MH = mental health, PF = physical functioning, RE = role emotional, RP = role physical, SF = social functioning, VT = vitality.

## 11. Discussion

The goal of this study was to explore the effects of bundled care during the perioperative period of coronary artery intervention (PCI), focusing particularly on postoperative complication prevention, patient recovery, and nursing satisfaction. For CAD patients undergoing PCI, the quality of perioperative nursing plays a crucial role in postoperative recovery.^[17]^ Recent studies have shown that traditional nursing models, which primarily focus on basic postoperative observation and interventions, often fail to integrate multidisciplinary nursing resources, resulting in a lack of personalized, systematic, and comprehensive care. As a result, bundled care, which integrates various nursing resources and provides comprehensive intervention, has gained increasing attention as an effective approach to improve patient outcomes and care quality.

The results of this study demonstrate that bundled care significantly improved postoperative outcomes for PCI patients, particularly in reducing postoperative complication rates, accelerating recovery, and enhancing nursing satisfaction. Specifically, the incidence of common complications such as puncture site infections, bleeding, and hematomas was significantly lower in the bundled care group compared to the routine care group. This outcome aligns with our expectations, as bundled care involves rigorous monitoring and timely interventions during the postoperative phase, reducing the risk of complications and promoting better recovery. These findings are consistent with those of Zhang et al (2024),^[18]^ who observed that nursing interventions can effectively reduce the incidence of bleeding and infection following PCI, highlighting the high application value of this care model in minimizing postoperative complications.

Furthermore, patients in the bundled care group exhibited significantly faster recovery, particularly in the restoration of early mobility and self-care ability. The accelerated recovery was demonstrated by early activity guidance on the first day after surgery, followed by functional rehabilitation training starting on the third day, which considerably shortened hospitalization. The bundled care group had a significantly shorter hospital stay compared to the routine care group, further improving patients’ quality of life. This finding is supported by Ribeiro et al (2023),^[19]^ whose research indicated that fine-tuned postoperative care management improves patients’ ability to self-care and shortens recovery periods.

In terms of nursing satisfaction, the bundled care group exhibited higher satisfaction, particularly in postoperative pain management, psychological support, and personalized care. Patients reported receiving comprehensive attention and care, particularly in terms of psychological support and health education, which helped them approach the recovery process more positively. This finding aligns with previous studies, such as Li et al (2022),^[20]^ who noted that systematic nursing interventions not only improved physiological recovery but also enhanced psychological comfort and overall nursing satisfaction, emphasizing the close relationship between nursing quality and patient satisfaction.

Importantly, the implementation of bundled care significantly reduced postoperative complications, especially among high-risk patients, such as those with diabetes or hypertension, who are more prone to complications due to lower immunity and slower recovery. Bundled care’s focus on dynamic monitoring and adjustment of blood sugar and blood pressure, along with personalized care plans, greatly reduced the risk of complications in these patients and accelerated their recovery. This effect is consistent with existing literature, highlighting the unique advantages of bundled care for high-risk patients.

Overall, the results suggest that Bundle care is effective in reducing postoperative complications, accelerating recovery, and improving nursing satisfaction in PCI patients, demonstrating its potential for perioperative management. However, the study has limitations. First, due to its retrospective and single-center design, there is a risk of selection bias, even though we employed PSM to reduce confounding and improve baseline comparability. Additionally, the relatively small sample size may have limited the statistical power to detect significant differences in some outcomes, particularly those with clinically meaningful but statistically insignificant trends. These results should therefore be interpreted with caution. Future studies should adopt prospective, multicenter, randomized controlled designs with larger cohorts to increase external validity and statistical robustness. Moreover, the specific components within Bundle care require further evaluation and refinement, as the optimal intervention mix may differ across patient subgroups with varying health statuses, comorbidities, and risk profiles. Future research could focus on personalized care models that tailor Bundle care strategies to individual patient characteristics.

## 12. Conclusion

Finally, the effectiveness of Bundle care depends not only on the expertise of nursing staff and multidisciplinary team collaboration but also on patient adherence and engagement. Strengthening staff training, improving patient participation, and enhancing interdisciplinary cooperation will be important areas for future research and clinical practice. Overall, under the sample size and design limitations of this single-center retrospective study, our findings suggest that Bundle care may contribute to improved postoperative recovery and nursing satisfaction in PCI patients. However, further prospective, large-scale, and multicenter studies are required to verify these results and confirm the model’s generalizability and long-term impact. These results provide preliminary support for the clinical application of Bundle care, but its broader implementation should proceed cautiously, guided by additional evidence.

## Author contributions

**Conceptualization:** Huarong Tan.

**Data curation:** Huarong Tan.

**Formal analysis:** Huarong Tan.

**Investigation:** Huarong Tan.

**Methodology:** Huarong Tan.

**Resources:** Huarong Tan.

**Visualization:** Huarong Tan.

**Writing – original draft:** Huarong Tan.

**Writing – review & editing:** Huarong Tan.
